# Detailed temporal structure of communication networks in groups of songbirds

**DOI:** 10.1098/rsif.2016.0296

**Published:** 2016-06

**Authors:** Dan Stowell, Lisa Gill, David Clayton

**Affiliations:** 1 Machine Listening Lab, Centre for Digital Music, Queen Mary University of London, London, UK; 2 School of Biological and Chemical Sciences, Queen Mary University of London, London, UK; 3 Max Planck Institute for Ornithology, Seewiesen, Germany

**Keywords:** animal communication, Poisson process, point process, linear–nonlinear Poisson, communication network, social network analysis

## Abstract

Animals in groups often exchange calls, in patterns whose temporal structure may be influenced by contextual factors such as physical location and the social network structure of the group. We introduce a model-based analysis for temporal patterns of animal call timing, originally developed for networks of firing neurons. This has advantages over cross-correlation analysis in that it can correctly handle common-cause confounds and provides a generative model of call patterns with explicit parameters for the influences between individuals. It also has advantages over standard Markovian analysis in that it incorporates detailed temporal interactions which affect timing as well as sequencing of calls. Further, a fitted model can be used to generate novel synthetic call sequences. We apply the method to calls recorded from groups of domesticated zebra finch (*Taeniopygia guttata*) individuals. We find that the communication network in these groups has stable structure that persists from one day to the next, and that ‘kernels’ reflecting the temporal range of influence have a characteristic structure for a calling individual's effect on itself, its partner and on others in the group. We further find characteristic patterns of influences by call type as well as by individual.

## 1. Introduction

Many animals exhibit group calling behaviour. Patterns of calling are observable phenomena which reflect individual state and are dependent on behavioural context [1,2]. Understanding the dynamics of vocalization patterns within groups is an important growing topic in animal behaviour [2–5]. However, analysing the structure of the communication network in a group of animals presents a challenge which goes beyond that of analysing calls of isolated individuals or pairs, because multiple influences converge on an individual in parallel, making it harder to characterize causal connections.

In this study, we introduce a model-based method for characterizing the temporal and network structure of interactions between calling individuals from the timing of call events. The paradigm was originally developed in computational neurology for analysis of spiking neural networks [6,7]. We adapted the method for the case of animal calls and applied it to data from groups of domesticated zebra finch (*Taeniopygia guttata*), a communal songbird that is the subject of much current research [8,9]. With this approach, we were able to represent zebra finch communication networks in a compact model whose attributes reflect fine details of timing and influence strengths between individuals in a group, yielding a new data-driven perspective that complements other approaches based on acoustics, neurology or ethology, and provides a useful visualization tool.

Before describing our study and analysis, we first wish to set our analytical approach in context by discussing methods for modelling animal vocalization sequences, in particular their applicability to vocalizations in groups.

## 2. Modelling the processes that generate animal vocalizations

Researchers analyse animal calling patterns in order to understand the processes that generated them, whether their focus is on intra- or interindividual mechanisms. A general paradigm with strong mathematical support is to choose a family of probabilistic generative models that might generate the phenomena of interest, and then to use model selection and/or parameter fitting to decide which model from that family best matches the data.

A good example of this is Markov modelling. A Markov model generates the next symbol in a sequence stochastically but with limited memory: a *k*th-order Markov model chooses the next symbol conditional on the most recent *k* symbols and independently of all previous history. Markov modelling has been applied widely to animal communications [4]; once vocalizations have been reduced to symbol sequences, data fitting can determine the transition probabilities between symbols, as well as the model order, i.e. the length *k* of the ‘memory’ [10]. A Markov model is usually an extreme simplification of the presumed underlying biological process and neglects important aspects such as call timing. Because of this, a Markov model is unable to model some notable aspects of vocalization such as ‘bursty’ call patterns. However, it is a broadly useful tool. Extensions of this approach augment the model to include unobserved state (the *hidden Markov model* long used for speech recognition [11,12, ch. 17], the structured repetition of symbols (the *semi-Markov model* [13,14]) or time gaps between events (the *Markov renewal process* [15–17]). For our present purposes, an important consideration is that Markovian models do not adapt readily to the case where multiple influences converge on an individual in parallel. It is possible to construct a Markov model for the sequence of events emitted by a group as a whole. However, the system still ‘remembers' only the most recent *k* calls, irrespective of whether they come from socially significant others (e.g. a breeding partner) or from socially insignificant individuals.

**Figure 1.**
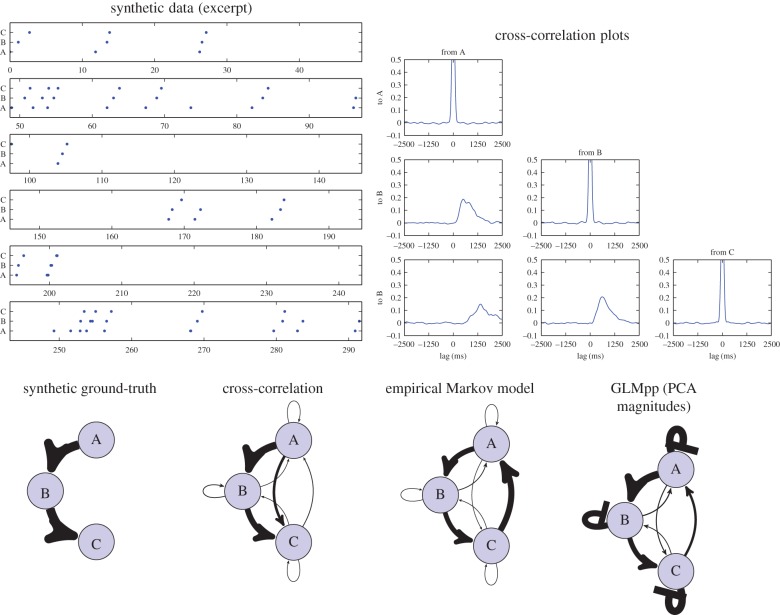
A synthetic example of indirect causation. A sequence was generated using a simplified A → B → C causal model, and then analysed using standard methods and our proposed method. The generation procedure was deliberately designed as not a perfect match to any of the analysis models, so as not to privilege any of them. We show an example timeline of events, along with the cross-correlation plots, and then the influence strengths recovered using each method summarized as a social network diagram. Arrow thicknesses indicate the relative strength of influence (or transition probability for the Markov model); arrows represent excitatory influences, while flat-headed arrows (the self–self loops in our analysis) represent inhibitory influences. Note that the values recovered by each method are different in kind and have been rescaled separately for each of the network plots. Cross-correlation analysis recovers most of the influences/independences but tends to recover false-positive connections for the indirect link A → C (see the lower-left panel of the cross-correlation plots). A simple Markov model recovers influences without timing information, and in this case also adds a connection from C back to A to take the place of the baseline event calling rate of A. Our proposed method recovers a good match for the network structure as well as timing information. It adds self-inhibitory feedback on B and C to account for the fact that in this test case a call by A leads to no more than one call by B (and likewise for B → C). For further details of this synthetic example, see the electronic supplementary material. (Online version in colour.)

Cross-correlation analysis can be used to analyse relative timing, but is not derived from a generative model: it is descriptive rather than inferential. A particular set of cross-correlation statistics may be compatible with multiple hypotheses about the underlying process (Markovian or otherwise). Cross-correlation is an appealing alternative to standard Markov modelling, because it gives some characterization of the time gaps between events, not just the event sequences. Studies based on cross-correlation typically probe for significant patterns but do not attempt to give a formal model that could have generated those patterns [2,18]. As one example of potential issues with cross-correlation, a causal network with a chain structure such as A → B → C may well create indirect cross-correlation phenomena from A → C, even where there is no direct causal link (figure 1), which would result in a clear instance of the maxim ‘correlation is not causation’.

The presence/absence of causal links can be probed through measures of *Granger causality* [19, §9.13] for influences with linear effects. Information theoretic measures can also be used, and are not limited to linear relationships: in particular *transfer entropy*, a measure of the conditional mutual information between one time series and another, is related to Granger causality and has been used to characterize the information transmission between neurons in a population [19, §9.13]. We do not pursue these approaches here since, as with cross-correlation, they do not provide a generative model.

**Figure 2.**
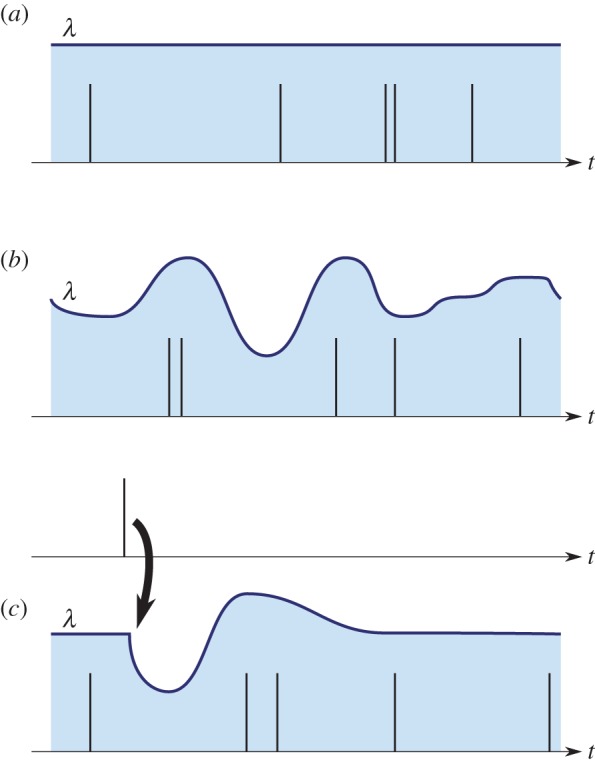
Schematic of processes generating events: (*a*) homogeneous Poisson process, (*b*) inhomogeneous Poisson process and (*c*) inhomogeneous Poisson process in which all changes in rate are due to the external influence of stimulus events. In each panel, the rate parameter for the process (*λ*) is shown as a filled curve, continuous in time, and an example sequence of events sampled from the process is shown as a sequence of spikes. (Online version in colour.)

A probabilistic model that is directly applicable to events on a continuous timeline is the *Poisson process* [20,21]. At its simplest, the (homogeneous) Poisson process outputs events stochastically but at a constant rate, meaning that the event times are random but there is a constant expected number of events per unit time (figure 2*a*). Most Poisson processes of interest are inhomogeneous, having a rate that can change over time (figure 2*b*). This Poisson process model can represent a single stream of events, but in order to capture interactions between individuals or between call types, we need to augment it with coupling such that calls from one individual can modulate the rate of calling of another (figure 2*c*). This will be achieved through influence *kernels K_ij_* whose effect is that a call from each individual *i* results in a modulating ‘wave’ of influence on the rates of each other individual *j*. The instantaneous firing rate for individual *j* is given by a *linear–nonlinear* link function, meaning that influences are linearly summed and then passed through a nonlinearity:
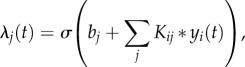
where we have used *b_j_* to represent the constant base rate of calling for *j*, *K_ij_* the influence kernel from individual *i* to *j*, * the convolution operation, and *y_i_*(*t*) the sequence of events emitted by individual *i* represented as a spike train. The function *σ* is a nonlinear mapping which can be freely chosen within certain constraints; these include that the function must be monotonic and non-negative [6]. In this work, our attention will be on the characteristics of the kernels *K_ij_*, which capture both the strength of influence from one individual to another and its temporal characteristics (both excitatory and inhibitory).

Note that we will include reflexive influences (where *i* = *j*) in our analysis. The feedback effect of an individual on itself may include self-listening, but could also account for a refractory period such as for drawing breath. For model-fitting purposes, it is important to include all known causal antecedents, including an individual's own calling history.

Such a point-process model can be fitted to data by maximum likelihood [6] after which the *K_ij_* reflect both the typical time gaps between events and the typical sequencing of one event after another. These coupled processes are not necessarily Markovian, and the modelling focus is slightly different: instead of the turn-by-turn sequencing which underlies Markovian models, the emphasis here is on separate processes each generating calls, happening in parallel, and these processes can mutually influence one another.

In this study, we wished to use exactly these point-process models, with interaction kernels, to elucidate the temporal structure of networks of calling birds. We have multiple motivations for doing this. The first is that the information from the fitted model may yield information similar to that which has previously been derived through cross-correlation [2,5], such as the strength of pairwise influences, but with more robustness to common-cause confounds and other issues discussed above. A second is that the fitted model may yield more finely nuanced information, and information tied to an explicit model: for example, one individual may have a suppressive effect on its neighbour at some timescales, and an excitatory effect at other timescales. Another is that a fitted model can be used to make further inferences about new datasets, for example, to predict whether communicating partners are paired or unpaired. Another is that since the model we fit is generative, it can be used to generate new synthetic sequences having the same network characteristics, which could be used for stimuli in future studies.

To this end, we conducted one study with a group of female zebra finches in a standardized context, and one reanalysis of existing data from a mixed-sex group of zebra finches in a different and varying context.

## 3. Material and methods

### 3.1. Data collection

Four adult female zebra finches (at least 90 days old, with wild-type plumage) were selected from an aviary of the QMUL animal facility. The four birds were housed together in a flight cage with free access to food and water, in a room separate from the main aviary. The group of four birds was housed together for more than two weeks before the recording sessions. The birds were kept on a 12 L/12 D cycle (7.00–19.00), and the room temperature was 20–21°C.

To perform the recordings, each bird was transferred to an individual cage (of size approx. 40 × 35 × 45 cm) with free access to food and water, and remained in visual and auditory contact with the other birds (at a distance of about 2 m). Birds were kept in the individual cages for just over 1 h d^–1^ (approx. 8.00–9.00) for recordings before returning to the group cage.

The solo cages were arranged in a square pattern so that all birds were approximately equidistant. Since we intended to investigate calling patterns as a function of bird identity, we avoided the potential confound of physical location by placing the birds in cages in different orders each of the 3 days, choosing the ordering by taking three rows from a four-by-four Latin square.

Audio was recorded during these 1 h sessions with four focal microphones (AKG C451B), one directed at each cage. All audio signals were recorded together onto a Zoom H6n multitrack sound recorder to ensure that the recordings were temporally synchronized. Recordings were made at 96 kHz sample rate and 16 bit depth. The first day of this sound recording protocol was used as a test run and for acclimation, and data were not analysed. The second and third days were taken forward for annotation.

We used a cross-validated semi-automatic process to label the audio events in the recordings. In a first pass, we applied automatic event detection to locate the beginning and end of events, using energy-based detection applied to spectrograms after performing median-filtering. Spectrograms were trimmed to the frequency region of interest (0.5–20 kHz). The four channels of the recording inevitably contained large amounts of ‘crosstalk’ as the protocol was designed so that the birds could clearly hear each other. Hence we used the median spectrogram across all channels as a background against which to judge the signal energy. Regions of high energy exceeding a minimum duration (8 ms; two spectrogram frames or more) were taken forward as candidates to the second stage.

The second stage of processing was manual refining of the detections. Each 4-channel spectrogram was divided into 1 s chunks (with an overlap of 50%) and the proposed annotation superimposed. Annotators were then shown the 1 s chunks in a random order, so that any variation in attention of the human annotators would not systematically vary across the recording duration. For each channel, annotators could listen to the audio and view spectrograms, confirm or reject the detected events and label them as calls or other noises (such as wing flaps or cage sounds). They could also label errors such as the merging or splitting of events, or misaligned event boundaries. Two separate annotators manually processed all of the candidate annotations. We used two paid annotators who were both PhD students with critical listening skills (audio engineering backgrounds). These annotators were trained by the first author, using excerpts from the pilot session recordings as examples. Consensus decisions were accepted, and deviations from consensus were resolved by the first author. Finally, the first author listened through to the full recordings with the annotations superimposed, as a check for any remaining anomalies.

For the present study, we then extracted the calling times for those events labelled as zebra finch calls. In this standardized recording environment, our female birds did not use a wide variety of call types (cf. [8]), predominantly the ‘Tet’/‘Stack’ type with a very small number of ‘Distance’ calls (note that female zebra finches do not sing). Therefore in order to provide a clear analysis, we did not split calls into different categories. This dataset of call times we refer to as *zf4f* (see ‘Data accessibility’, point (i)). In this dataset, there were around 2800 calls in each hour-long session (around 12 calls per bird per minute).

### 3.2. Analyses

We analysed the call timing information using the GLM point-process model of [7], with specific configuration described in the electronic supplementary material (hereafter referred to as *GLMpp*). Our main unit of analysis was each 60 min recording session as a whole, although we also analysed each 15 min segment separately, to investigate whether there was continuity or change of communication patterns throughout a session. We determined from preliminary tests that 15 min was the smallest region we could use to have enough calls for a stable analysis.

We used penalized maximum-likelihood (maximum *a posteriori*) optimization to fit the model parameters to each dataset. We fitted two models to each of our 60 min sessions—one combining influences in additive fashion, one in multiplicative fashion—and used an odds-ratio test to select the most appropriate model. In all cases, the additive model was favoured. More details on the modelling are given in the electronic supplementary material. Our source code to perform analyses and generate figures is available online (see ‘Data accessibility’, point (iii)).

The GLMpp model, after fitting to a dataset, yields a continuous curve (a ‘kernel’) for each directed pairwise influence. For four birds, this gives 16 kernels, four of which represent self–self influence and 12 of which represent self–other influence. In order to make quantitative and visual comparisons, we summarized kernels in two main ways. Firstly, we aggregated self–self and self–other kernels separately to look for general tendencies that might emerge independent of bird identity. Secondly, we applied principal components analysis (PCA) to the kernels to project them into a two-dimensional space summarizing the main dimensions of variation among kernels. Our model for each kernel has many degrees of freedom, to allow for many possible shapes, and this high dimensionality could lead to overfitting problems when performing tests of similarity/difference between kernels. We therefore used the two-dimensional PCA projection when testing for consistency within/between sessions and within/between social relationship types, as well as for visualization. When applying PCA we did not centre the kernels (by subtracting the mean), which has the advantage that the axis origin retains its meaning as zero influence. In the PCA space, therefore, the distance from the origin can be considered a simple summary of the magnitude of influence represented by a given kernel.

To test for consistency between sessions, we took these PCA magnitudes from the influence kernels, and measured the Pearson correlation from one day to the next. Since self–self and self–other kernels were different in kind (having positive and negative peaks, respectively), we analysed them separately, giving *n* = 12 for self–other and *n* = 4 for self–self. To investigate whether any correlation was due to individual identity, to physical location of the cage or to chance, we measured the correlation using four different ways of matching one day up with the next day. These four matchings were the combinations given by a Latin square: one matching compared the same individual across days, one matching compared the same physical location (and microphone) across days, while the remaining two were null matchings with no meaningful interpretation.

To test for consistency within sessions, we took the self–other magnitudes for each 15 min segment, and measured via Pearson correlation how strongly a segment could predict the immediate next segment. For each 1 h session, this gives three sequential pairs of segments.

All of the above analysis was applied to our own *zf4f* dataset. We also applied the analysis to a subset of the call data from [2] in which interindividual call timings had been studied in a more complex group setting. These groups were made up of freely behaving zebra finch males (four) and females (four), whose vocalizations were individually recorded via backpack microphones, over a period of three weeks. During this time, all birds were able to interact physically, and to engage in various activities, while pair-bonding, nesting and breeding progressed. We used GLMpp as an alternative to the cross-correlation analysis of Gill *et al.* to investigate group calling behaviour on a finer timescale, including an investigation of self–self calling patterns. As all individuals in the analysed data formed pair bonds, we not only distinguished between self–self and self–other interactions as in *zf4f*, but we also separated out the self–partner interactions to investigate any consistent patterns emerging specifically within pairs. We were provided with the data for trial II, days 1, 7, 11, 18—the same days as displayed in fig. 5*a* of [2]—which span the different breeding stages of that group (see ‘Data accessibility’, point (ii)). Each session was just under 4 h long.

To evaluate formally whether specific types of interaction kernels differed according to the nature of the social connection (self–self, self–partner or self–other), we applied a *multiple response permutation procedure* (MRPP), a permutation test for between-group differences based on distances [22, ch. 2]. For this, we used the *vegan* library in R v. 3.0.2 [23], using Euclidean distance, and stratification according to the identity of the listener. As the number of items in each category was small, we carried out all MRPP tests in the two-dimensional PCA-reduced space to avoid overfitting. From this test, we quote the ‘chance-corrected agreement’ statistic, which summarizes within-group consistency (by measuring the average within-group distance between items after normalization by the overall average distance between items).

In the data of Gill *et al.* [2], calls were categorized into types. We applied GLMpp to all calls together (as with our own data), but since this dataset contained a larger number of calls across various types, we also explored splitting the data according to call type so that each pairwise interaction could have a different kernel for each call type. Note that Gill *et al.* use five call categories, which implies that for each directed pair we must fit 25 different kernels rather than just one, and so in practice we found that the full analysis by call type was less stable due to data sparsity issues. Hence the recovered per-type kernels showed larger variances and the results are in some cases less clear-cut than for the broader aggregate models. We applied MRPP *post hoc* to these kernels, to quantify the separability of kernel types in specific cases.

For further comparison of methods, we also analysed this dataset using a cross-correlation method: the peri-stimulus time histograms (PSTHs) of Gill *et al.* [2] but taking all calls pooled together irrespective of type. This enabled us to inspect whether the call timings considered as a whole contain signatures of social context, under each analytical approach. The PSTH method yields a correlation index which is a measure of the tendency for each individual to call during the time window 50–500 ms after a stimulus call, normalized against the base rate of calling, and is further described in [2].

## 4. Results

### 4.1. All-female group (*zf4f*)

We first show results from our recordings with four female zebra finches in a standardized context. The model-selection test consistently selected the model in which influences were combined by addition (rather than multiplication). This is in contrast to Pillow *et al.* [7] applying the same method to an analysis of spiking neurons, which selected the multiplicative model.

**Figure 3.**
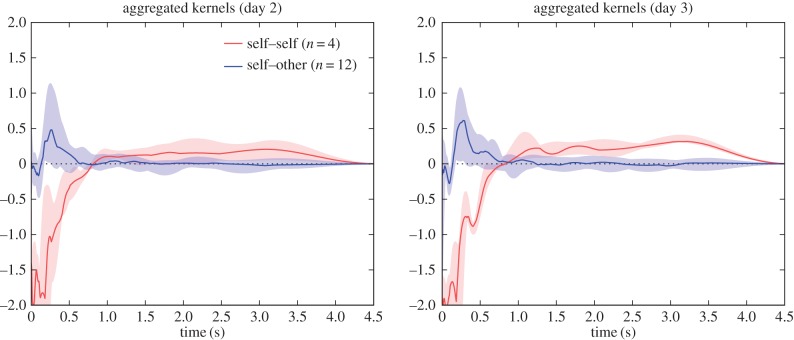
Aggregate view of the influence kernels recovered from our two study sessions with four female zebra finches (for details on how to read these plots, see text).

**Figure 4.**
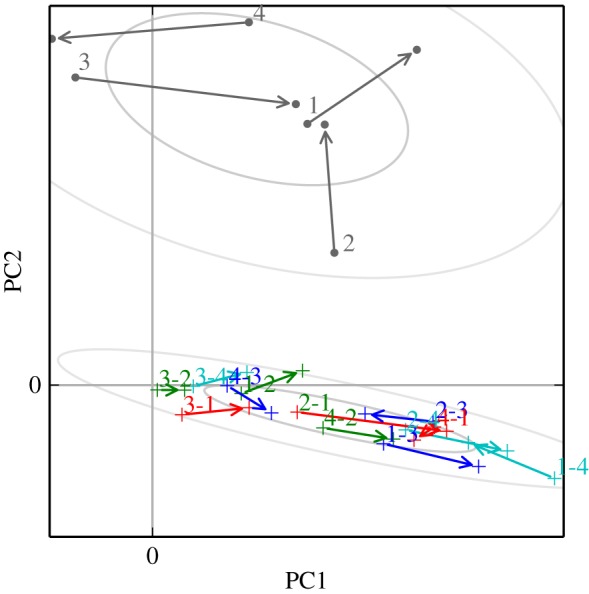
Principal components plot of kernels recovered from *zf4f* data. Arrows connect the kernels for each directed pair, between the two days studied. Ellipses show the 50% and 95% probability regions for Gaussian fits, for the self–self (upper, dots) and for the self–other (lower, plusses) datapoints. These Gaussian ellipses give a visual indication of the groupings evaluated in the MRPP test. The number labels indicate the individuals involved in each influence kernel. (Online version in colour.)

We found that influence kernels exhibited consistent temporal characteristics and broadly consistent magnitudes, and that these differed very strongly between self–self and self–other interactions (figures 3 and 4). The confidence intervals, despite aggregating over individuals, were relatively narrow with little overlap between self–self and self–other. This was confirmed by the MRPP test (within-group agreement 41.3%, *p* = 0.0001).

*How to read figure 3 plots*. The kernel plots in figure 3 and elsewhere summarize the kernels across the entire communication network, grouping the kernels according to whether they represent a self–self (red lines) or self–other (blue) connection. For each directed pair of birds, we inferred a single kernel curve; these plots show the median curve, and the 5–95 percentile range, across all possible pairs of individuals in the group. Hence the filled regions largely indicate the extent of variation among the network connections. The time on the *x*-axis can be thought of as similar to the ‘lag’ in cross-correlation. The *y*-axis can be thought of as the ‘excess calling rate’ caused by a stimulus (although this interpretation is complicated a little by the nonlinearity; see the electronic supplementary material for detail). Imagine that a bird emits a call at time zero. The plot then shows the effect of that call over the next few seconds, increasing and/or decreasing every bird's tendency to call. Unlike a Markov model, the call at time zero is not considered to lead to a single call that happens as a consequence of it: another bird might call once, twice or more during the period in which it is strongly stimulated by the call at time zero. (In practice, the strong inhibition we see—the strong negative peak for self–self interactions—often suppresses multiple responding.) A flat kernel with a value of zero would correspond to statistical independence, indicating that one bird had no effect on the calling rate of the listener. The influences from multiple individuals are added together by the listener before being passed through a nonlinearity; the main effect of the nonlinearity (for interpretive purposes) is a soft-thresholding to prevent the rate going below zero. For self–self kernels, the lag includes the lag due to the duration of the call itself (median duration 0.1 s).

**Figure 5.**
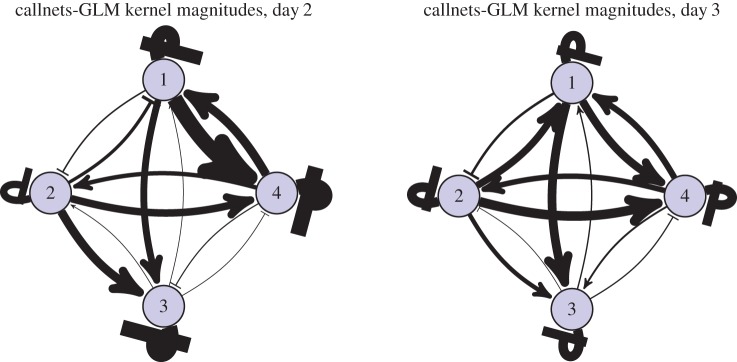
Influence strengths, plotted as a social network. Standard arrows indicate kernels whose peak values are positive (excitatory), while flat-headed arrows indicate kernels whose peak values are negative (inhibitory). In this case, all the self–self arrows looping back are inhibitory. Note that this view emphasizes the magnitudes while suppressing the temporal structure recovered using our model. (Online version in colour.)

The self–other kernel magnitudes from one day to the next were strongly predicted by individual identity (*p* < 0.001), and not by physical location or by the null combinations (table 1). Physical location did not yield any observable effect (*p* = 0.79), having a correlation compatible with the null permutations. Thus, we attribute the variation in interindividual influence strengths (figure 5) to individual identity. In this study, we did not find that self–self kernel magnitudes were predictable from one day to the next: Pearson correlation was 0.48 (*p* = 0.52), which fell within the range of the null permutations.

**Table 1.** Predictability (Pearson correlation) of kernel magnitudes, from one day to the next, measured under four different permutations for aligning the two days. The four permutations correspond to four rows of a Latin square, one of which matched individuals across days, another which matched physical cage locations across days and two null permutations having no meaningful interpretation.

data permutation	self–other (*n* = 12)	self–self (*n* = 4)
individual	0.82***	0.48
location	−0.09	0.37
null 1	−0.41	0.60
null 2	0.11	0.29

***p < 0.001.

When analysing the sessions in 15 min segments, we found consistency but also variation in the self–other influence strengths. Values did not remain constant but often were generally variable with characteristic typical magnitudes, and were moderately predictable from the immediate preceding segment (Pearson correlation 0.37, *p* = 0.0013), confirming that the between-day consistency can be observed on the finer scale of 15 min segments despite the observable variation. On this timescale, we also observed consistency in the self–self peaks, at a similar moderate level (Pearson correlation 0.46, *p* = 0.025).

Individuals exhibited a pattern of strong self-suppression immediately after calling and for around the next 0.8 s, followed by a slight positive effect thereafter. By contrast, self–other interactions showed a consistent positive peak at around 0.25 s, before decaying to around zero at 0.7 s, indicating a consistent characteristic timescale for calls that occur in response to the calls of others. In this group of females, although the network influences showed consistency, there was no evidence for strong structure of the network such as a hierarchy (figure 5).

### 4.2. Reanalysis of Gill *et al.*

**Figure 6.**
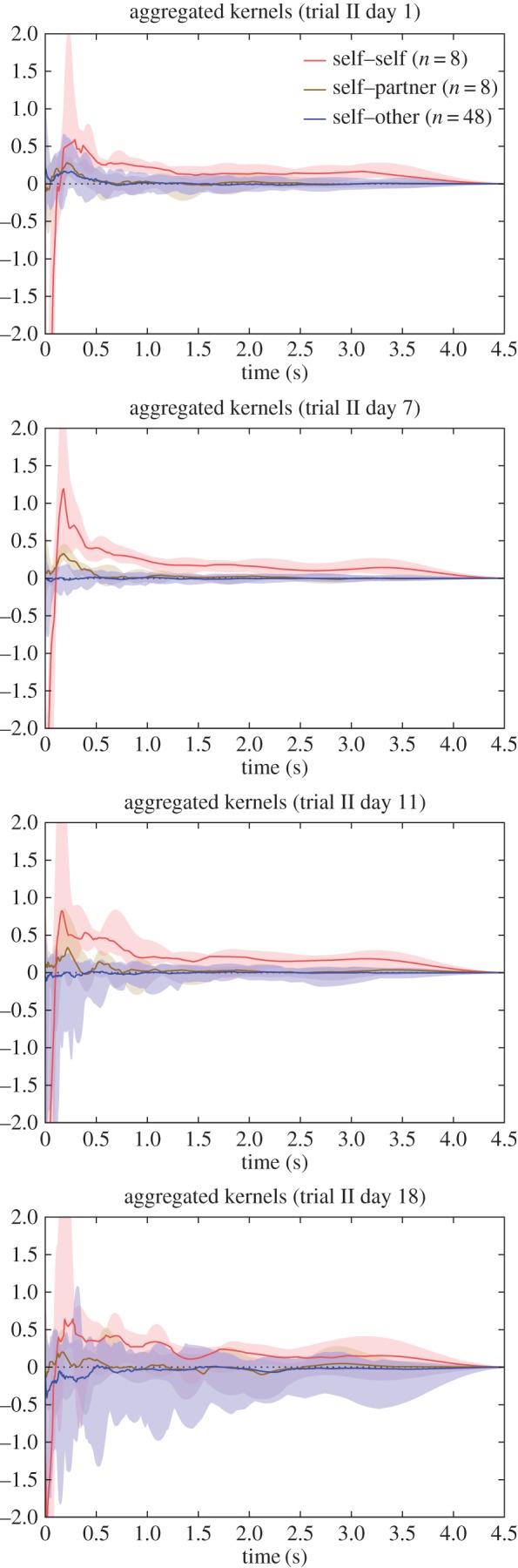
Aggregate kernels as in figure 3 but for the dataset of Gill *et al*. Note that the ‘self–partner’ category was labelled retrospectively, according to the pair bonds that eventually formed. The pairings had typically not yet formed on the first day.

The groups studied in Gill *et al.* were in a very different environment—mixed-sex and larger groups, with the ability to physically interact, to undertake nesting and breeding. This is reflected in some notable differences in the typical influence kernels compared against those from the *zf4f* data (figure 6).

**Figure 7.**
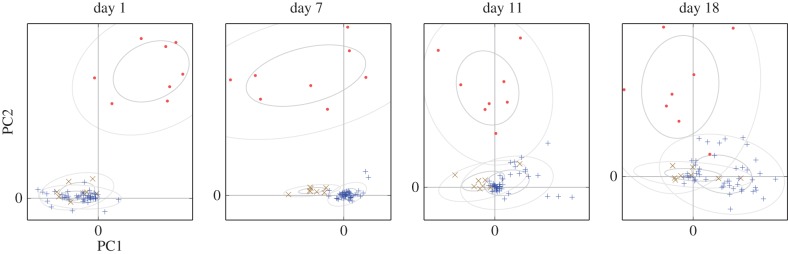
Principal components plots of kernels recovered from each day of the Gill *et al*. data. Ellipses show the 50% and 95% Gaussian probability regions for the three types of connection: self–self (dots), self–partner (crosses), self–other (plusses). (Online version in colour.)

**Figure 8.**
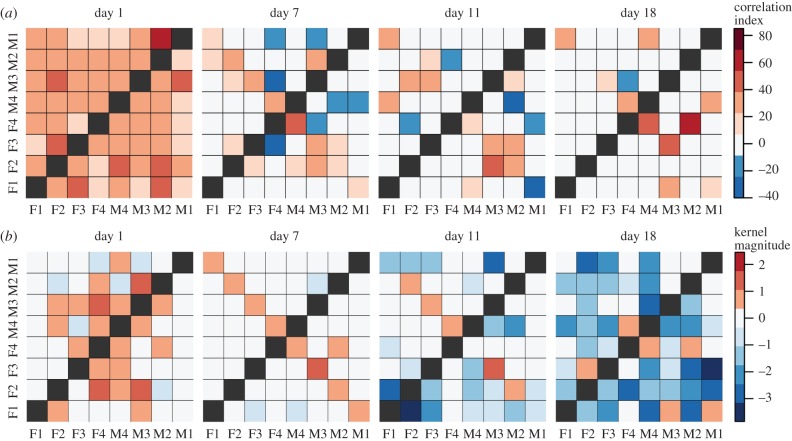
Interindividual influence strengths for the dataset of Gill *et al*. considering all call types pooled together and analysed using cross-correlation (*a*) or GLMpp (*b*). In each matrix, individuals are arranged so that the cells on the counter-diagonal are the intra-pair influences (e.g. F1 to M1). Self–self influences are omitted for visual clarity (black squares on diagonal). For the GLMpp analysis, PCA kernel magnitudes are plotted, with polarity used to indicate whether each kernel's main peak is excitatory or inhibitory.

Again the different kernel types show continuity over multiple sessions. Here, however, we observed specific developments in the communication network as the pairs progressed through different stages of bonding and breeding. On the first day, when pairings were yet to stabilize, there was little difference between self–other and self–partner influence (note that the ‘self–partner’ category was labelled retrospectively, so in the early days it indicates *eventual* partners). As partnerships formed and developed they took on specific within-pair communication characteristics: by day 7, when nest material was provided and many of the birds were involved in nest-building, communication showed a specific self–partner peak with a timescale around 0.2 s, while the typical influence of non-partner birds (self–other) had reduced down close to independence (figures 6 and 8). In other words, group communication was dominated by within-pair patterns. In the later days, this structured communication subsided somewhat, although self–partner influences continued to be stronger than self–other influences. As before, the self–self kernels were strongly differentiated from all the other kernels (MRPP agreement 22.6%, *p* = 0.0001). The self–partner kernels were not at all separable from the self–other kernels on day 1; they became strongly distinctive on day 7, and remained separable in subsequent days but with decreasing clarity (figure 7 and table 2). We see that the data from day 1 did not show a strong signature that might have predicted the eventual pairings; rather, the trend seen visually is that the ‘self–partner’ interactions were very similar to the self–other interactions before pairs had formed, but then reached high levels with the established partner while the interactions with non-partner individuals decayed down to weak or inhibitory influences.

**Table 2.** Within-group agreement for self–partner versus self–other influence kernels, for each day of the Gill *et al*. data, with *p*-values.

day	MRPP agreement (%)	*p*-value
1	0.2	0.2359
7	35.7	0.0001
11	8.5	0.0008
18	5.8	0.0067

**Figure 9.**
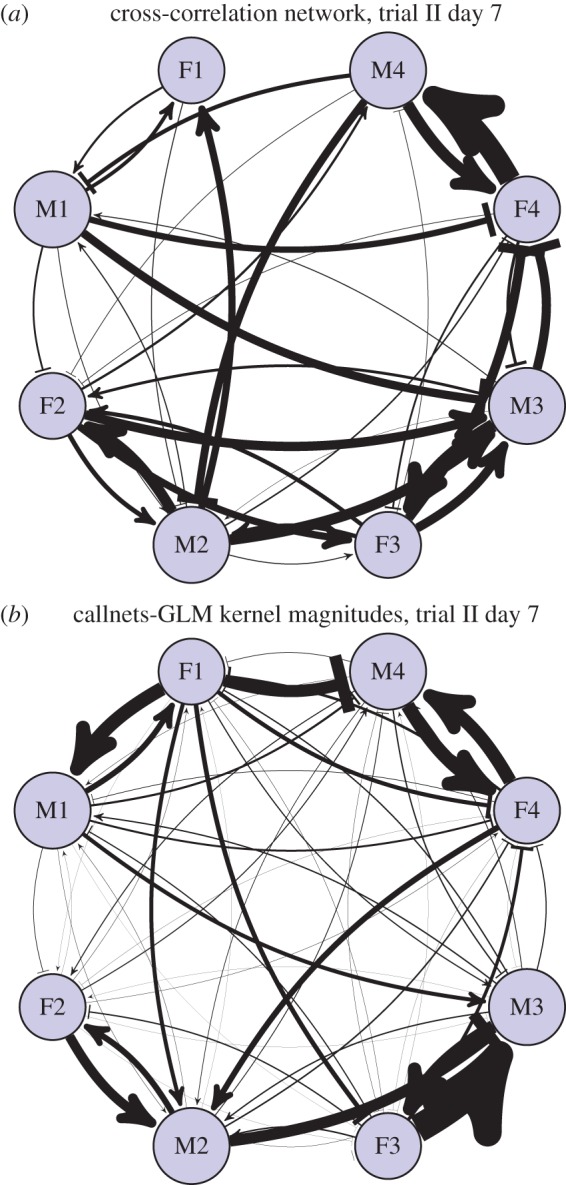
Network plot of interindividual influence strengths for the dataset of Gill *et al*. on day 7, considering all call types pooled together and analysed using cross-correlation (*a*) or GLMpp (*b*). See also figure 8. (Online version in colour.)

Visualizing the influence strengths makes clear the social network structure that was evident on day 7. Even considering only the magnitude (and not the temporal structure) of influences, and considering all call types pooled together, the network structure showed an observable signature under both the cross-correlation and GLMpp analyses, though more clearly for GLMpp (figures 8 and 9).

We note that the self–self influence kernels recovered from these data were rather different from those in the *zf4f* recordings. There was in both a strong immediate self-inhibition effect, but in the present case this was followed by a self-excitation at around 0.2 s which was not observed in the *zf4f* data. The implication of short-term self-excitation is that calls are being emitted in bursts or sequences. The median self–self influences showed bumpy multi-modal curves which suggested that there might be further structure in the patterns of typical gaps in the sequences, or that the aggregate plots were merging together different kernels which each had differing timescales. We inspected the detail of individual kernel plots and found that the latter was not the case: there were no observable individual differences in overall self-excitation timescales.

**Figure 10.**
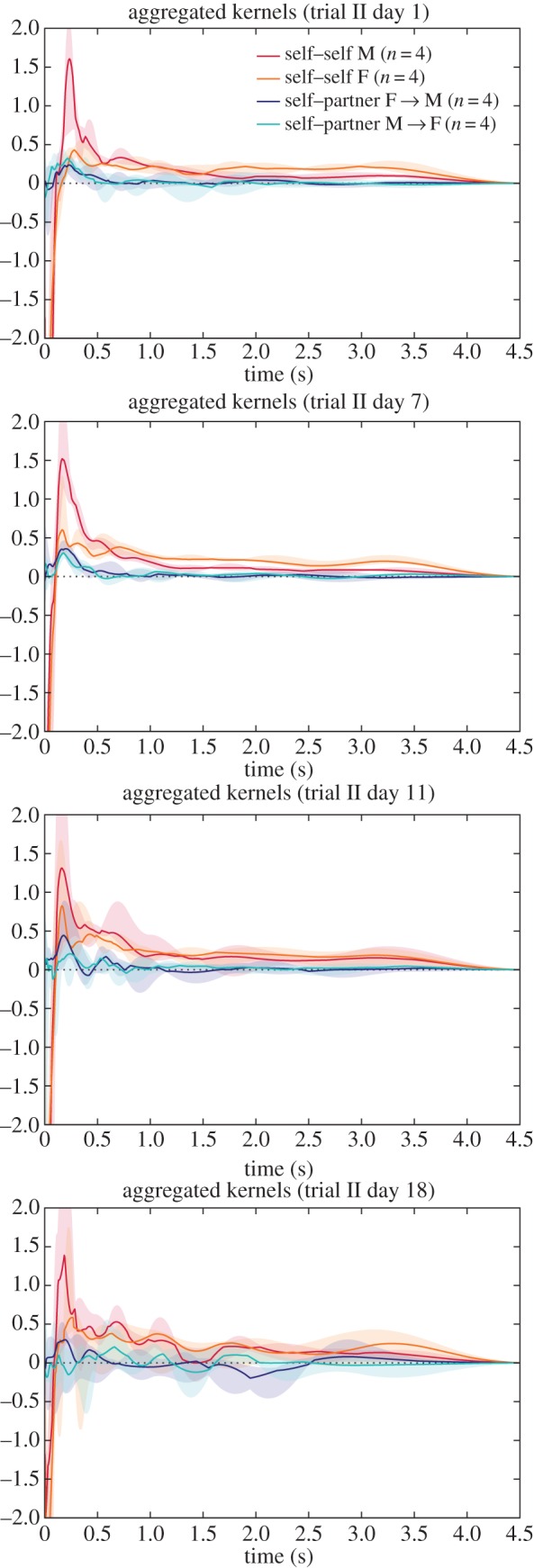
Aggregate kernels for the dataset of Gill *et al*. but showing only the within-pair interactions (self–self and self–partner) and further breaking them down by sex.

When inspecting the differences between males and females (figure 10), we found this short-term peak in self-excitation was seen more strongly in males. The pattern became clearer when inspecting the kernels derived after separating the calls into behavioural call types. It was observed to lie predominantly in Cackle calls, specifically in an individual following a Cackle with another Cackle (figure 12). The kernel plots broken down by call type exhibited more variance than the main plots due to data sparsity, but nevertheless only the Cackle → Cackle self–self influence showed this strong rapid peak (at around 0.15 s), and this was consistent across the different days analysed.

Cackles explained one component of the multi-modal self–self kernels. However, we could not conclude that the overall self–self kernel was explained as merely a sum of unimodal influences varying by call type, as the broken-down kernel plots did not generally resolve to simpler structure.

**Figure 11.**
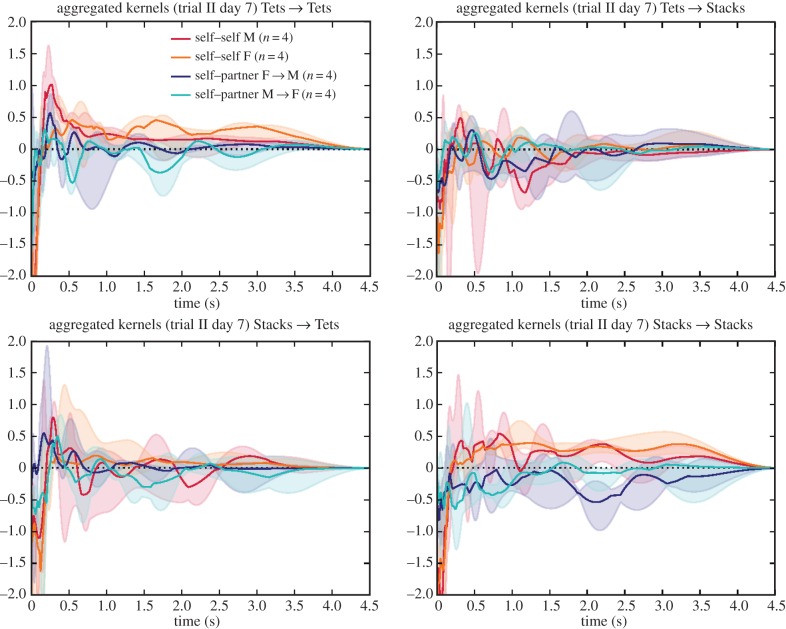
Aggregate kernels specifically for the ‘Tets’ and ‘Stacks’ of Gill *et al*. and their interactions, on day 7.

Other aspects of the kernels broken down by call type confirm the observations of Gill *et al.* In many cases, the strongest effect of a particular call type was to induce responses of the same type, but with some influences from one call type to another. Around day 7, we observed a tendency for Stacks or Tets from a female to induce Tet responses in the male partner (figure 11), as was also remarked upon in [2], and for the male Tets to have a notable self-excitatory peak. By day 7, birds were largely in the nest-building and later nesting stages (fig. 2 of [2]).

**Figure 12.**
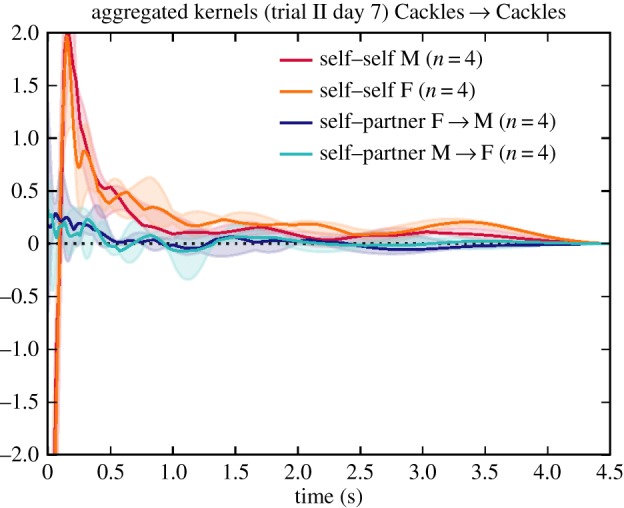
Aggregate kernels specifically for the ‘Cackles’ of Gill *et al*. on day 7.

Note that dividing the calls into five types gives a 25-fold increase in the number of influence kernels to be recovered, which may lead to data sparsity in some cases. This is visible in the increased variance of the per-type kernel estimates (figures 11 and 12). For this reason, we will only discuss per-type kernels in which we observe clear patterns, or indicative patterns which triangulate against observations made in related work [2,24]. Quantitative analysis of the separability of self–partner Tet and Stack calls found a significant but moderate distinction between their interaction kernels on day 7, and no separability on the other days (table 3).

**Table 3.** Within-group agreement for each day of the Gill *et al*. data, to measure the mutual distinctiveness of four specific influence kernel types: Tet–Tet, Tet–Stack, Stack–Tet, Stack–Stack (cf. figure 11).

day	MRPP agreement (%)	*p*-value
1	−1.6	0.623
7	7.7	0.029
11	−2.8	0.638
18	−3.2	0.664

## 5. Discussion

When animals interact in groups, multiple influences converge on each individual in parallel and the effects of these influences depend on social context. In groups of zebra finches, we found temporal interaction patterns that were consistent: they persisted over time according to sender/receiver identity, and they had characteristic structure depending on the nature of the social bond (self–self, self–partner, self–other; male–female, female–male) and on contextual status such as breeding stage. These effects were observable considering all call types pooled together, without having to separate calls into subcategories, which has not been possible in previous work.

We characterized these communication networks by fitting a simple general-purpose model which takes account of the parallel known influences converging on an individual. The model is flexible enough to represent a wide range of pairwise influence patterns (kernels), including kernels which show patterns of suppression *and* excitation together, depending on relative timing.

When reanalysing call data previously presented by Gill *et al.* [2] in which cross-correlation had been used to identify interactions, we were able to confirm many of the observations, but demonstrated in more detail the temporal structure of interaction patterns, including self–self interactions. In some social contexts (particularly nest-building), within-pair influences dominate and calling patterns become much less strongly influenced by extra-pair group members. The structure of the communication network changes qualitatively through the different stages of zebra finch breeding activity.

We also found qualitative differences in communication influence patterns between our female-only group and the mixed-sex group of [2]. Note that the groups differed in various ways (presence/absence of mates, ability to interact physically, group size, backpack microphones, aviary environment, handling) and so we do not here attribute the differences between the two studies to specific contextual parameters; that remains for future study. However, the per-sex and per-type plots strongly suggest that sex differences in timing patterns, and the presence of specific self–partner interactions, are the dominant factors in the differences seen between the two studies.

### 5.1. Zebra finch call types and their use in vocal interactions

Our reanalysis adds extra detail to the use of the different call types recorded and studied in [2]. For example, we quantify the separability of the Tet and Stack calls, not through acoustic analysis but through the distinctiveness of their timing influences (figure 11 and table 3).

Another example is the specific self-excitation pattern observed for Cackle calls (figure 12). The specific pattern we observed corresponds with behavioural observations in the literature: ‘[Cackle] calls are emitted in sequence either by one single partner (especially by the male when leading the nest search; Zann 1996a, b) or by both birds that are then performing soft duets using these calls in combination with Tet calls (Elie *et al.* 2010)’ [24, p. 301]. As well as the self-excitation pattern, we found a short-term self–partner excitation effect, corresponding to the duetting mentioned.

Elie *et al.* [24] take issue with the categorization of Tet/Stack calls used by Gill *et al*. [2] and Ter Maat *et al*. [18]. They propose that the ‘Stack’ of Gill *et al.* is not the ‘Stack’ which Zann observed in wild zebra finches [8], but rather that it is a variant of the ‘Tet’. They argue that the ‘Tet’ and ‘Stack’ are used in very similar behavioural circumstances, and so should all be considered under the general category of ‘Tet’. On the other hand, their own acoustic analysis finds them to be similar but distinct clusters, and so they suggest they could be referred to as ‘Tet-M’ and ‘Tet-S’ to avoid confusion with the ‘Stack’ of Zann.

In this light, our analysis may help to illuminate whether the two categories annotated in [2] show different interaction patterns. The question is whether the two call types are behaviourally equivalent. If this were the case, we would certainly expect the Tet → Tet and Stack → Stack kernels to have similar characteristics. We might also expect the cross-type influence kernels (Tet → Stack and Stack → Tet) to be broadly similar. Note that we would not necessarily expect the cross-type kernels to look the same as the within-type kernels: for example, the cross-type kernels might show smaller influence in the hypothetical case that Tets and Stacks are behaviourally equivalent but emitted in different states of arousal, and therefore unlikely to happen in close temporal proximity.

Contrary to this hypothesis of equivalence, we found indications of differing influence kernels both within and between Tets and Stacks on day 7 (figure 11 and table 3), in which we saw that both Tets and Stacks from a female showed a tendency to inspire a Tet response from a partner within around 0.25 s, but this was not seen for Stack responses. Stacks and Tets also showed differing self-excitation patterns, indicating that the short-term sequencing of bursts had different character. We treat this as indicative only, as the measured distinction between Tets and Stack influence patterns was moderate and was only evident on the day of heightened self–partner interaction specificity. Our analysis does not disprove the claim of Elie *et al.* [24] that Tets and Stacks lie on a continuum and are used in similar situations. However it indicates that as well as having observable acoustic differences, Tets and Stacks may be used differently within communication interactions on the timescale of seconds.

Elie *et al.* [24, p. 300] describe the Tet call thus: ‘The Tet call is the most frequent vocalization as it appears to be produced in an almost automatic and continuous fashion when zebra finches move around on perches or on the ground. These ‘background’ Tet calls form an almost continuous hum and do not appear to produce a particular response in the nearby birds’. Contrary to this, we find that Tets do have an effect of inducing Tets from a partner on a specific timescale. In this, we concur with Gill *et al.* [2].

It is worth noting that in our presentation we have not focused on the resting ‘base rate’ of calling, which in our model is the component that causes birds to call in the absence of any stimulus. The base rates here took values of approximately ±0.15 per individual; the peak influence spikes were on a similar or larger scale and thus had a non-trivial effect compared against the base rate. A particular appeal of our modelling approach here is that it can identify components of influence even in the presence of a base calling rate.

### 5.2. Reflections on GLM point process methodology

The GLM point-process method we have used is relatively generic—it can be applied to neurons as much as to calling animals—and as we have shown, it is flexible enough to capture a variety of phenomena which are pertinent to the understanding of animal calling interactions. It can capture specific timescales and strengths of influence, both positive and negative and mixed, between individuals, including asymmetric influences (A → B can be different from B → A), and provides a useful representation separating specific influences out from the calling base rate. It can reproduce bursty/sequential calling phenomena in individuals or groups. The method has a number of advantages over cross-correlation analysis. Directed causation is directly modelled rather than implicit. (This should not be interpreted as claiming that the method uncovers the full set of factors having causal influence on an individual: the model abstracts over physiological detail, and characterizes the relative strengths of the causal factors proposed by the analyst.) Multiple convergent influences are simultaneously modelled as well as a default base rate. Thanks to this, spurious links due to common-cause effects are less likely to occur. One example of the benefit of this is seen in our analysis by Gill *et al.* in which, even with all calls pooled together rather than separated by type, the effect of social structure is seen much more clearly than via a more conventional method (figure 8).

The method is not specialized for strict sequencing: for example, if birds always emitted exactly three Cackles in a sequence, this could not be modelled. In fact, this limitation is in common with the standard Markov model. Future modelling advances may add useful generalizations, for example, the incorporation of hidden state variables. Strict sequencing can be described in a hidden Markov model or a semi-Markov model, but those are in general suitable only for independent individuals and not a good fit for situations with multiple influences within a group.

The point-process model we have described here is closely related to a set of self-stimulating statistical models called ‘Hawkes processes’ [25,26]. For example, the method of Hall & Willett [25] has an appealing property of ‘streaming’ performance, meaning that the network characteristics can adapt continuously through time as the network evolves. However, their model has important limitations which the GLMpp model does not. It does not incorporate the nonlinearity which allows for flexibility and ensures that the model remains meaningful in the presence of negative influences (which otherwise could yield meaningless negative calling rates). More importantly, under their model every link in the network must have the *same* kernel shape and only the magnitudes can vary. We have demonstrated clearly in this work that zebra finch calling networks require, at minimum, different kernels for self–self, self–partner and self–other interactions, which have dramatically different shapes.

Our method models each calling individual as an inhomogeneous Poisson process, where the changes in calling rate are due to external influences. An alternative approach is to model each individual (slightly more simply) as an inhomogeneous Poisson process (figure 2*b*), and then look for correlations among their inferred underlying calling rates. An advantage of that approach would be to accommodate smooth modulations in the base rate of calling; however, this comes at the significant cost of probing the modelled rates only indirectly for evidence of causal influence (much as in cross-correlation analysis), rather than directly fitting a causal model to the observed data.

Looking slightly more broadly, there are some existing methods in the animal behaviour literature that have rough analogies to our approach, but using different types of behavioural data. Psorakis [27] use spatio-temporal proximity of animals as indirect indicators of affiliation, which are then used to infer a social network graph. Note that the method there can only infer undirected (symmetric) connections between pairs of individuals, not directed connections as in the case of calling patterns here. Another rough analogy is with Nagy *et al*. [28], who infer pigeon hierarchy from delays in flock movement responses. In that case, the observed data are continuous movement data and temporal cross-correlations in movements are the clues used, instead of discrete events, to infer networks.

The data size requirements of the GLMpp model are reasonable for our purposes, as indicated by our ability to recover stable repeatable influence kernels with 15, 60 or 240 min of data. (See also the electronic supplementary material for a simulation test on data size requirements.) The approach requires the same amount of data as does cross-correlation. We note that dividing the calls down by call type as well as by individual can lead quite quickly to data sparsity issues. This is seen in the slightly rough nature of the median kernels and higher variance in figure 11 versus figure 6. This is of course true for other analysis methods as well. The GLM analysis has been applied to datasets having hundreds of neurons, and so it has the ability to scale to larger groups than we have studied [7].

The computation required to fit these models is larger than to run a cross-correlation test. In our largest data fit, analysing one of the almost 4 h sessions from [2] and breaking each of the eight individuals' calls down into five types—yielding a 40-by-40 fully connected network of influences to infer—this took 7 h on an ordinary laptop (2.6 GHz Intel i5, four cores). Analysis can be made faster if some connections can be ruled out *a priori*, such as the influence from whines to distance calls, which we know from behavioural observations, previous work [1,2] and the present study do not show any notable influence.

The paradigm that we have applied in this study is relatively abstract and generic. This has two implications. First, it means that the fitted models can and should be compared against behavioural observations and against any more customized behavioural and/or physiological models for the species being studied, to explore the convergence of these different sources of evidence. Second, it means that this approach is not limited to songbirds, nor to communal species, and may find application in other taxa such as mammals or territorial songbirds.

The GLMpp model is generative, which allows for interesting experimental designs that can be considered in future, such as generating large numbers of novel group call sequences as stimuli, synthesizing background ‘crowd’ sounds, or creating group interactions in which live individuals interact in real time with automatic conversational participants.

## Supplementary Material

Supporting Information

## Data accessibility

Our call timing data are publicly available via Figshare:

(i) *zf4f*: https://dx.doi.org/10.6084/m9.figshare.1613791
(ii) Gill *et al.* data subset: https://dx.doi.org/10.6084/m9.figshare.2061327
(iii) Our source code to perform analyses and generate figures is available online via: https://github.com/danstowell/callnets-glm.
