# Supplementary material for: Detailed temporal structure of communication networks in groups of songbirds
Source: J R Soc Interface. 2016 Jun;13(119):20160296. doi: 10.1098/rsif.2016.0296 (PMC4938092; doi:10.1098/rsif.2016.0296)
Supplement: Supporting Information [file rsif20160296supp1.pdf]

Supporting Information for: Detailed temporal structure of communication networks in groups of songbirds

Dan Stowell, Lisa Gill, David Clayton

GLM point process model fitting

Our use of the GLM point process model broadly follows that of Pillow et al.[1]. Here we describe specific configuration choices and adaptations we made to the model.

Choice of nonlinearity

In the GLM point process model, the instantaneous firing rate $\lambda_j(t)$ is given by a *linear-nonlinear* link function, meaning that influences are linearly summed and then passed through a nonlinearity:

$$\lambda_j(t) = \sigma\left(b_j + \sum_j K_{ij} * y_i(t)\right) \tag{1}$$

where we have used b_j to represent the constant base rate of calling for j, K_{ij} the influence kernel from individual i to j, $*$ the convolution operation, and $y_i(t)$ the sequence of events emitted by individual i represented as a spike train.

The function σ is a nonlinear mapping which can be freely chosen within certain constraints; these include that the function must be monotonic and non-negative [2]. If σ is the exponential function, then this has the effect of transforming (1) so that the influences combine in multiplicative fashion rather than additive. If σ is the rectifier function $\max(0, \cdot)$, this preserves linear additivity except that negative values are clipped away. However, the rectifier function can be difficult to perform numerical optimisation upon, because a large part of its domain gives zero gradient, and often a smooth approximation is used in its place, which we describe shortly.

Within this simple model, in which animal behaviour is embedded in rather abstract form, it is not immediately clear which nonlinearity should be chosen. (Note that the method is rather robust to misspecification, meaning that under mild conditions it yields consistent kernel estimates even if the wrong link function is used [2, Section 5].) Hence we chose to use these simple nonlinearities and use model comparison to choose between additive and multiplicative models, as

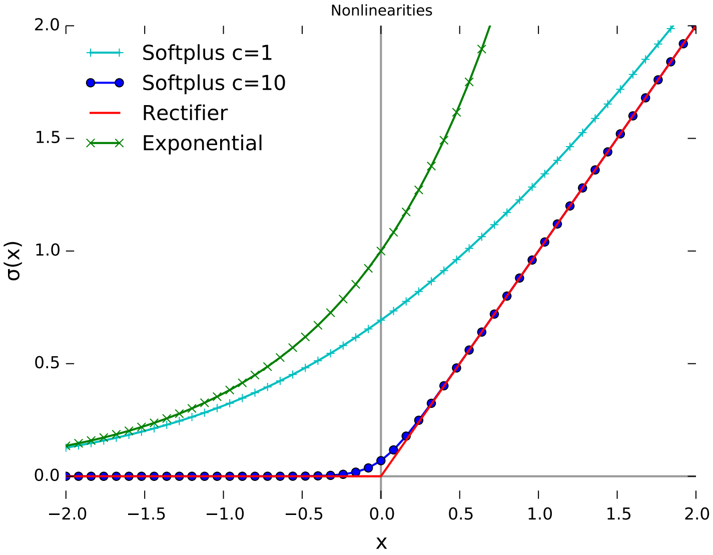

Supplementary Figure 1. Nonlinearity functions.

was done in [1]. In early tests we found that the strict rectifier function often failed in optimisation, and so in its place we used the smooth *softplus* function

$$\lambda(x) = \log(1 + \exp(cx))/c \tag{2}$$

where we introduced the constant scaling factor c to bring the function closer to the rectifier nonlinearity. The softplus function is not scale-invariant and so its effect (and hence the desirable choice of c) depends on the dynamic range of the data. We used $c = 10$, chosen to be large enough to bring the function close to a true rectifier effect without leading to numerical optimisation problems (Fig. 1).

When given an input of zero, our softplus nonlinearity yields a rate of around 0.07 (one call expected every 15 seconds). Negative inputs suppress the rate even further: in theory the rate never quite reaches zero, while with the machine precision used here, a zero rate of emission is produced from an input of around -4 or less. Positive inputs become increasingly close to the identity mapping; any input larger than around 0.3 is within 1 per cent deviation from identity.

Typical values of the base-rate parameter b in fitted models were approximately around ± 0.15. The inter-individual variation in b was smaller than the general scale of the influence kernels.

Kernel basis functions

The model works by parametrically combining a set of basis functions to create each kernel. This keeps the dimensionality of the model tractable while allowing for great flexibility in the range of kernels possible. We used a set of raised cosine

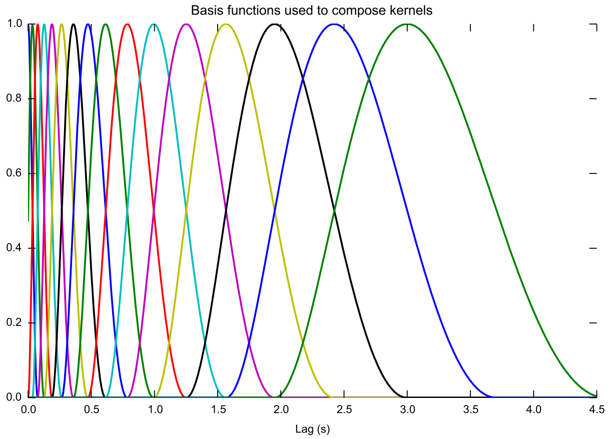

Supplementary Figure 2. The raised-cosine basis functions used to compose each kernel.

functions (Fig. 2), distributed with a logarithmically-warped time axis to offer more detail at short time lags. In initial tests we first used 8 basis functions. We varied the degree of warping to inspect whether the choice of bases had a strong influence on the resulting kernel fits. We found that with 8 basis functions the placement of the bases had an observable effect on the undulations of the median fitted kernels, but if we increased to 16 basis functions the recovered kernels were much more stable to the exact choice of basis. We therefore used 16 basis functions for the main analyses. The same set of basis functions was used for all network connections, including self-self and self-other.

Regularisation

The model fitting method finds a single point estimate of the best fitting parameters. This is done by maximum likelihood in [1], but it can also be done by maximum *a posteriori* (MAP; also referred to as a type of penalised maximum likelihood) within the same framework [2]. It is widely known that maximum likelihood methods are vulnerable to overfitting in the presence of limited data. We therefore used MAP regularisation in the GLM model to help prevent overfitting at small data sizes and to stabilise the fitted parameter estimates. We used standard L_2 (Euclidean) regularisation when fitting the model, which is equivalent to a zero-centred Gaussian prior on the coefficients (Fig. 3). We did not regularise the parameter b—the fixed base-rate for each individual to emit an event in absence of stimulation—allowing it to take any value with equal prior probability.

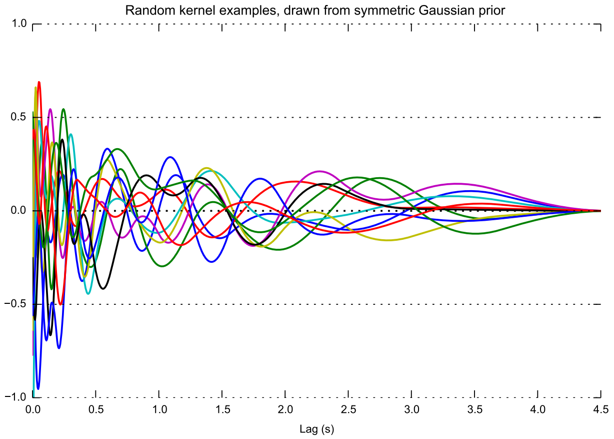

Supplementary Figure 3. Ten randomly-sampled examples of kernels from the prior distribution of the GLM model (i.e. without any exposure to data), to illustrate the range of kernels that can be fitted and also some qualities of the prior. Note the symmetry of the prior distribution around zero, and the relative smoothness of kernels at long lag times—because the basis functions are distributed exponentially with respect to lag time.

Principal components analysis of kernels

Here we give further details of the PCA analysis of recovered kernels, and show the principal component kernel shapes.

For each dataset the PCA projection was applied across all relationship types and all days pooled together, so that the two-dimensional space recovered was a common space representing the general variability of kernels recovered in each study. We did not centre the data (did not subtract the mean) so that the origin retained its meaning as independence. Since the polarity of the direction represented by each principal component is arbitrary, we chose to orient each of the principal components so that the average loading of each was positive.

Principal components were dissimilar across the two datasets (Fig. 4), reflecting the different tendencies discussed in the main text. The amount of variance explained by the first two PCs was 91.4% for *zf4f* but 67.2% in the Gill et al. dataset, likely reflecting the greater variety of social connections underlying the latter.

Synthetic A→B→C example and data size tests

The A→B→C synthetic example given in Fig. 1 of the main document was generated from a simplified model that is not an exact match to the GLM or MRP generative models, in order to provide a clear example of indirect causation and to examine how the various analyses behave. First, 'A' events were generated from a homogeneous Poisson process with fixed rate of 0.1 Hz.

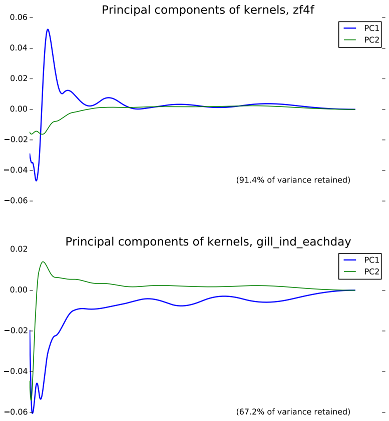

Supplementary Figure 4. Kernel shapes represented by the first two principal components in *zf4f* (upper) and Gill et al. (lower).

Then, for the two links in the causal chain, A→B and B→C, each possible source event generated a target event with a probability of 0.9, and if it did so, with a time gap drawn from a log-Gaussian distribution with log-mean 0.75 and log-standard deviation 0.5 (using the natural logarithm). The parameter values here were manually chosen to be on a similar timescale as zebra finch calls, but not intended to be a likely zebra finch scenario, rather to demonstrate the common-cause phenomenon. The generative model just described does not quite match the GLM point process model since no more than one event can be generated as a result of any other event, and there is no nonlinearity in the influence on rates. Nor does it match a pure Markov model: although each A→B→C sequence considered on its own is Markovian, the process can generate overlapping sequences meaning the overall sequence has no upper bound on its history dependence. This was therefore a useful test of how the different analysis procedures respond to data coming from a simple causal chain. Using this procedure, a timeline of length 96,000 seconds was generated.

For cross-correlation, we used a custom Python script to implement cross-correlation with a maximum lag of 2.5 seconds. Spike data were smoothed using a Hann window of duration 200 ms.

Data size tests In order to get a practical impression of the data size requirements of the GLM method compared against cross-correlation, we applied both methods to the synthetic A→B→C model while varying the number of data points fed into the analysis. This way we could guarantee that the source of data was a stationary system with unchanging characteristics, and inspect convergence as data size increased.

The analysis models are not designed to recover the same information, so they are not suitable for direct numerical comparison (e.g. mean squared er-

ror between models). We quantified the stability of each model via Spearman correlation against the largest fit (Fig. 5, right). Under the A→B→C model there are six inter-individual interactions to analyse, or three if undirected pairs are considered. We visualised the fitted parameters by allocating each of the three pairings to a colour (red/green/blue) and superimposing the three cross-correlation curves. The GLM model recovers six inter-individual kernels so we concatenated each kernel with its kernel in the opposite direction, to produce three shapes that could be colour-mapped in the same way.

We visualised the parameters recovered from the cross-correlation and GLM models, and also the GLM model without regularisation (i.e. maximum likelihood rather than maximum *a posteriori* fitting) (Fig. 5, left).

We found that cross-correlation and GLMpp converged at similar rates, reaching acceptable stability at around 1024 data points. As expected, the use of regularisation in the GLM model helped prevent overfitting at small data sizes, and to stabilise the fitted parameter estimates. We therefore used this same regularisation for all fits reported in the paper.

Simulating from the fitted models

Since our model is generative, we can draw newly simulated event sequences from the fitted models, which will thus be qualitatively similar to the original material. These sequences can be of arbitrary length and could even be generated in online (real-time) fashion. We can also use resimulation as another approach to inspecting and validating the fitted models.

As a simple illustration of this, we resimulated 3600 seconds of calls using the models fitted to the *zf4f* data. We resimulated the group (Fig. 7), and also we resimulated a single individual taken out of the group context, i.e. with only base-rate and self-self effects (Fig. 8). Between these two different simulations, the effect of group interactions was visible in the data: the same virtual individual produced calls at a rate of one per 14 seconds when simulated alone, and at a rate of one every 6.5 seconds when simulated in the group. (The real data contained a call from that individual every 4.6 seconds.) The resimulated group data replicates qualitative aspects of the real data (Fig. 6) such as the bursty communal calling, though appearing slightly less dense.

Re-running the GLM fitting procedure on the resimulated data for the group recovered kernels with the same shapes as in the source model (Fig. 10).

Further plots from datasets

For the *zf4f* dataset, overall calling rates per individual ranged from 468 to 888 calls per hour (Table 1), and rates exhibited some modulation across each hour (Fig. 9). A composite of all the influence kernels fitted to the dataset is shown in Fig. 11.

For completeness we show the fitted kernels for all possible call-type interactions, for a specific day of interest in the Gill et al.dataset (Fig. 12).

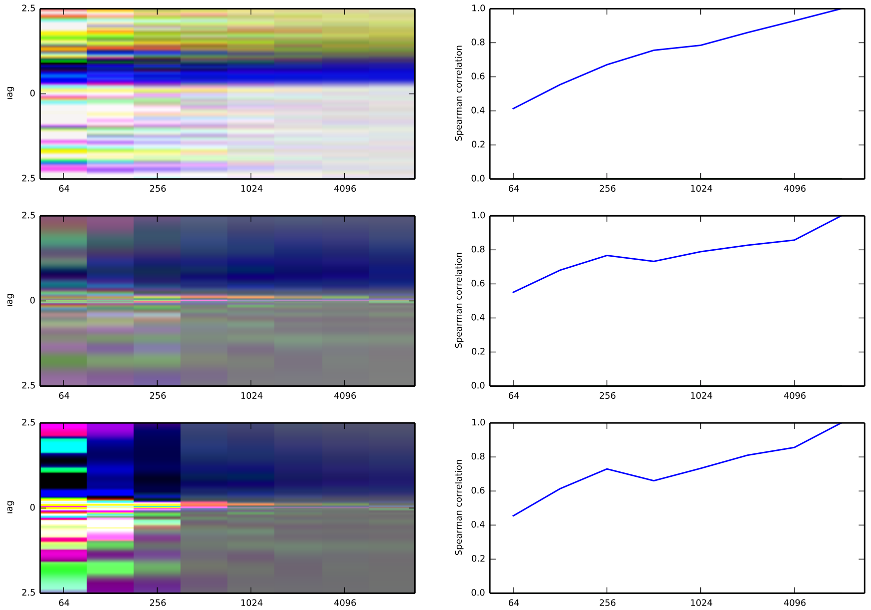

Supplementary Figure 5. Convergence characteristics of three models: cross-correlation (upper), GLMpp (middle) and GLMpp without regularisation (lower). In each case we provided the model with varying amounts of data sampled from the ABC synthetic model. For each model, we take the largest fit as the gold standard, and inspect how far the smaller fits deviate from it. This is summarised in the right-hand column by the Spearman correlation. The left-hand column shows a visualisation of the models at each step, to show more detail than the correlations. Within each visualisation, a column shows the estimated kernels (or cross-correlation curves) superimposed as channels of an RGB image, and then normalised for dynamic range. The amount of data used increases towards the right: of interest is at what left-right point the visual image stabilises. The lower plot appears bolder because the unregularised GLM tended to dramatically large parameter values when overfitting to small datasets. In the upper plot, the yellow band happens to reflect the false-positive A→C cross-correlation peak.

Supplementary Table 1. Total calls emitted by each bird in the *zf4f* recording study.

Individual	Calls in Day 2 session	Calls in Day 3 session
1	761	736
2	888	468
3	481	850
4	856	612

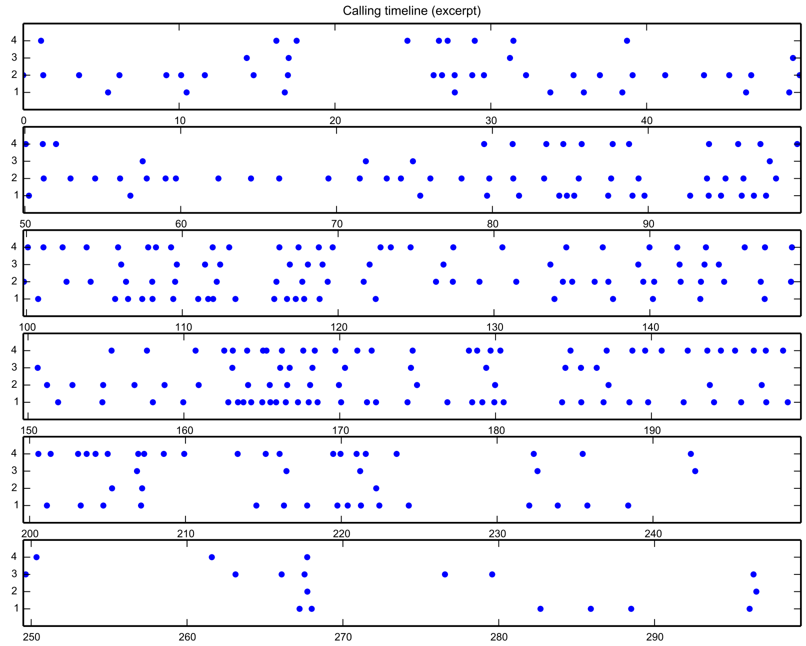

Supplementary Figure 6. Timeline plot of an excerpt from Session 2 of *zf4f*.

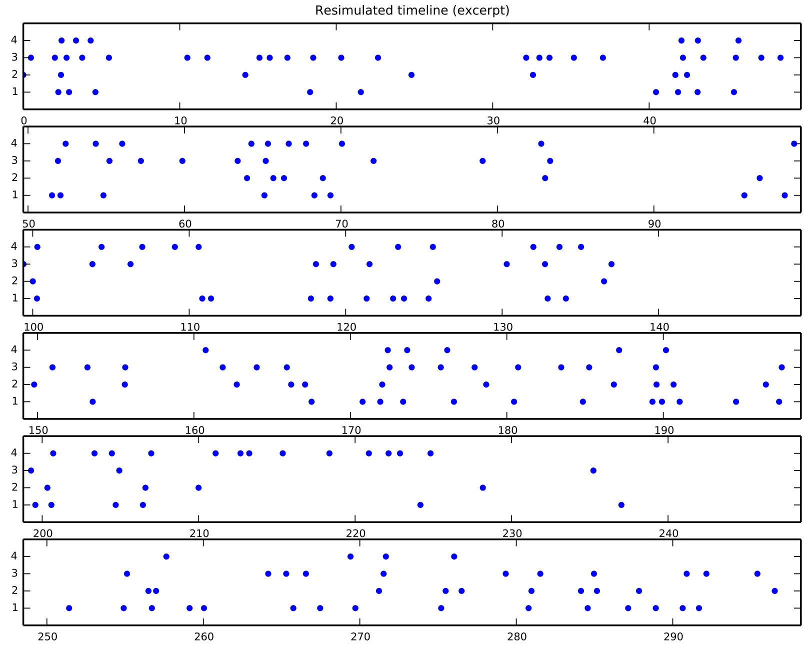

Supplementary Figure 7. Timeline plot of artificial data resimulated from fitted model for Session 2 of *zf4f*.

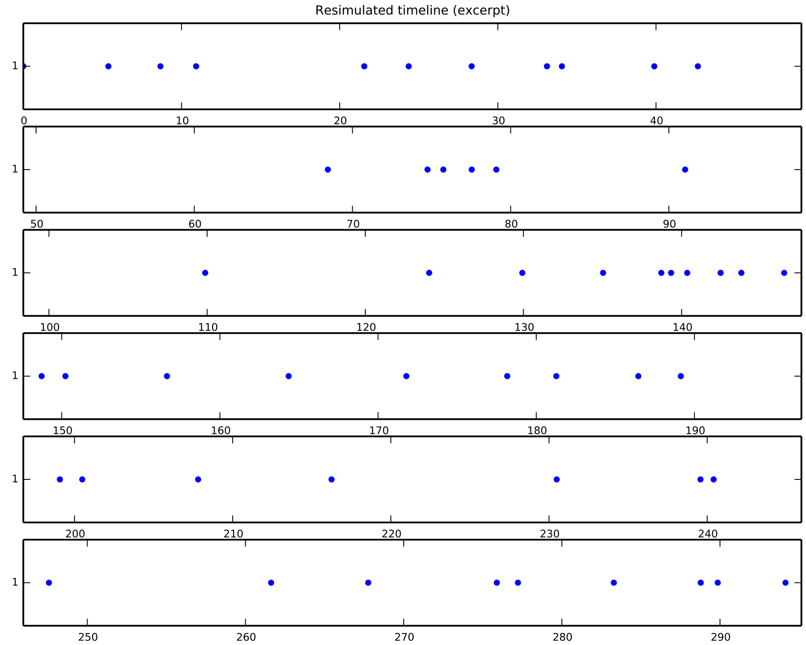

Supplementary Figure 8. Timeline plot of artificial data resimulated from fitted model for Session 2 of *zf4f*, in this case with only one virtual bird and no group.

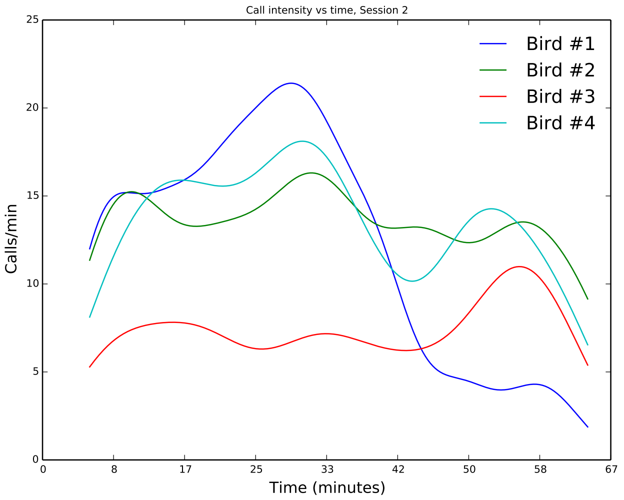

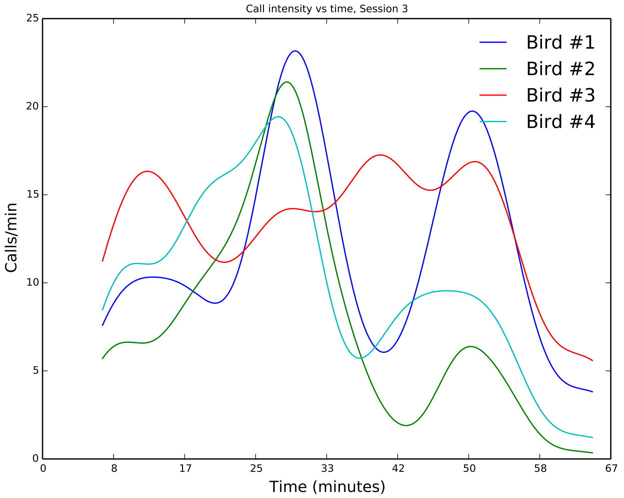

Supplementary Figure 9. Density plots of the calling rates of each bird in the *zf4f* recording study. Rates are calculated from call times using kernel density estimation with a bandwidth of 15 seconds.

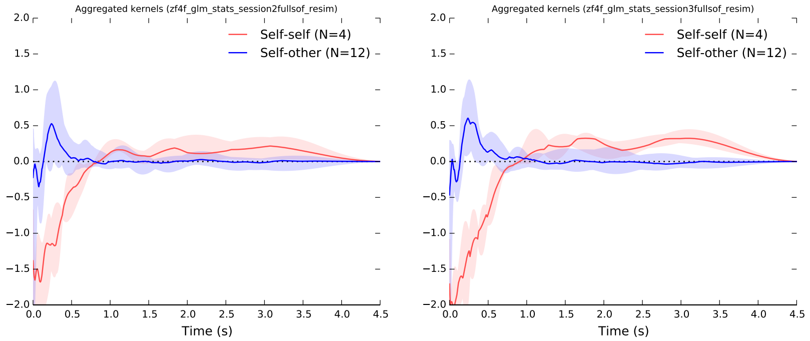

Supplementary Figure 10. Kernels recovered from the resimulated data.

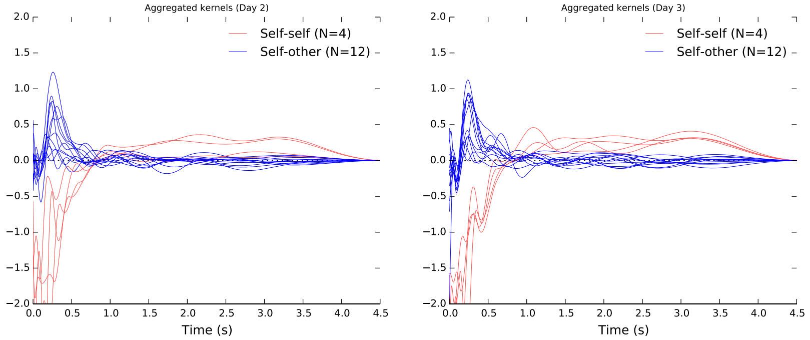

Supplementary Figure 11. Aggregate view of the influence kernels recovered from our two study sessions with four female zebra finches, as Fig. 3 in the main document but showing every one of the 16 curves fitted to each session, rather than confidence intervals.

References

1. Pillow JW, Shlens J, Paninski L, Sher A, Litke AM, Chichilnisky EJ, et al. Spatio-temporal correlations and visual signalling in a complete neuronal population. Nature. 2008;454(7207):995–999. doi:10.1038/nature07140.

2. Paninski L. Maximum likelihood estimation of cascade point-process neural encoding models. Network: Computation in Neural Systems. 2004;15(4):243–262.

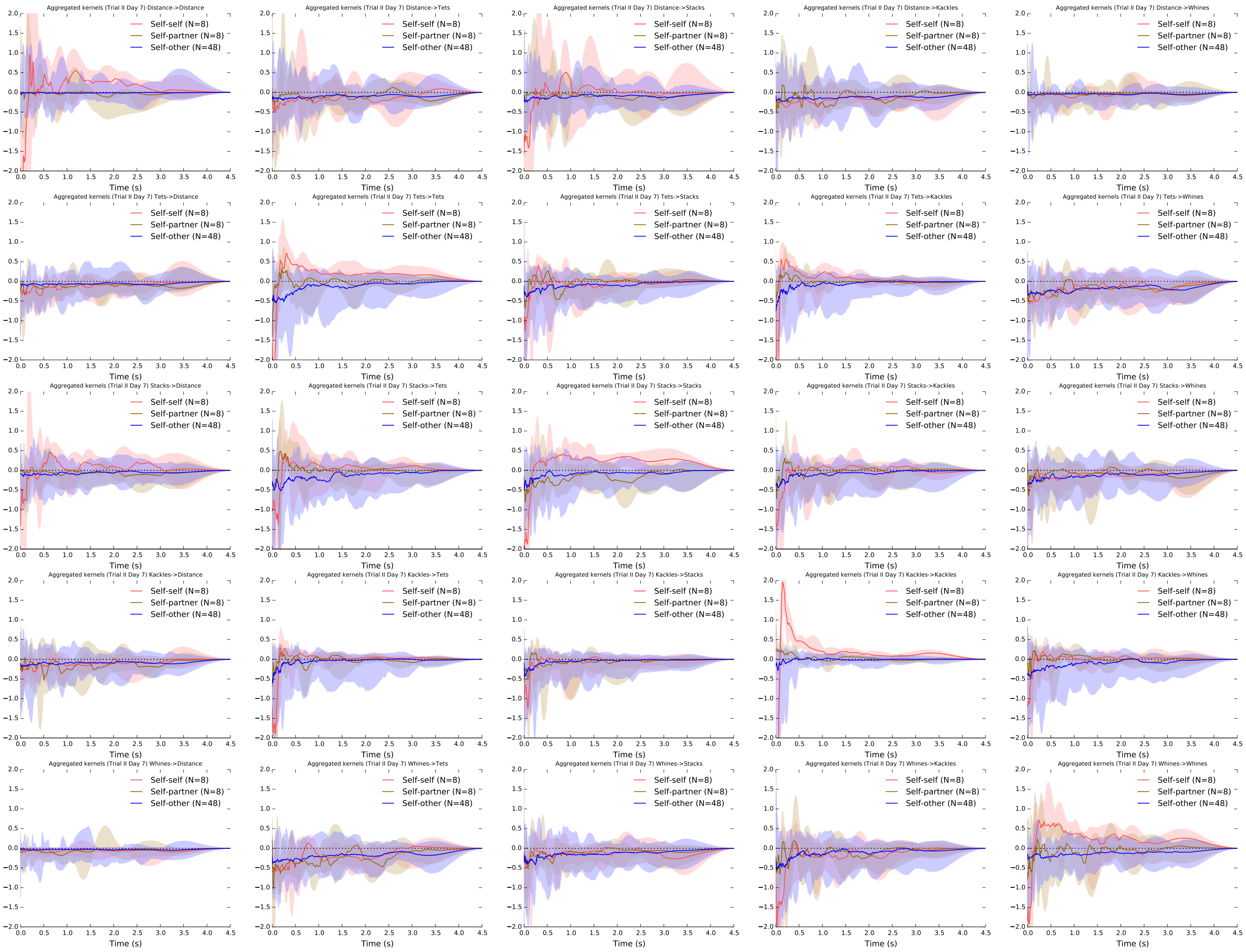

Supplementary Figure 12. Aggregate kernels for all possible call-type interactions, for the data of Gill et al.on day 7.
